# Perioperative immune checkpoint inhibitors in elderly patients with resectable NSCLC: a systematic review and meta-analysis

**DOI:** 10.3389/fonc.2025.1589846

**Published:** 2025-09-17

**Authors:** Yue Cao, Yumeng Tian, Lin Li

**Affiliations:** ^1^ Department of Pharmacy, Beijing Health Vocational College, Beijing, China; ^2^ Department of Medical Oncology, Beijing Hospital, National Center of Gerontology, Institute of Geriatric Medicine, Chinese Academy of Medical Sciences, Beijing, China

**Keywords:** perioperative immunotherapy, resectable NSCLC, elderly patients, meta-analysis, pathologic complete response, event-free survival (EFS)

## Abstract

**Background:**

Perioperative immunotherapy has shown promising results in patients with resectable stage II-III non-small cell lung cancer (NSCLC). However, its benefits for the specific subgroup of elderly patients remain unclear. This study aims to evaluate the efficacy of perioperative immunotherapy in elderly NSCLC patients aged 65 and above, focusing on key metrics such as pathological complete response (pCR), event-free survival (EFS), and overall survival (OS).

**Methods:**

We conducted a comprehensive meta-analysis of randomized clinical trials that reported subgroup data on elderly patients regarding pCR rates and hazard ratios (HRs) for EFS and OS. Data were retrieved from PubMed, EMBASE, and proceedings of oncology conferences from January 2020 to June 2025.A fixed effects model was used for the meta-analysis. Aggregated pooled HRs for time-to-event outcomes (EFS and OS), odds ratios (OR) and risk ratios (RRs) for dichotomous outcomes (pCR) were calculated specifically for patients aged ≥65 years who received perioperative immunotherapy or placebo.

**Results:**

A total of 8 randomized controlled trials involving 1561 patients aged ≥65 years with resectable NSCLC were included. A significant benefit was observed in terms of pCR (risk ratio, 5.26; 95% CI, 3.54 – 7.82; I² = 0%) and EFS (HR, 0.64; 95% CI, 0.55 – 0.74; I² = 7%) for patients aged ≥65 years who received perioperative immunotherapy compared with placebo.

**Conclusion:**

Our systematic review and meta-analysis demonstrated that perioperative immunotherapy was superior to placebo in terms of pathological and event-free survival for patients aged ≥65 years. These findings provide age-specific evidence to inform precision decision-making for treating the elderly patients.

**Systematic review registration:**

https://www.crd.york.ac.uk/prospero/, identifier CRD420250654072.

## Introduction

Lung cancer poses a major public health challenge globally, being the foremost reason for cancer-related deaths, with non-small-cell lung cancer (NSCLC) comprising more than 80% of all lung cancer cases (1). The incidence of NSCLC is more prevalent among the geriatric population, with those aged 65 and above accounting for approximately 46.9%, and those aged 70 and above making up around 20% (2). There is no chronological age threshold to define an older adult. However, age 65 and above is commonly accepted as the chronological definition of an older adult, primarily because it aligns with the eligibility age for Medicare benefits. This criterion has been adopted by the National Comprehensive Cancer Network and other authoritative bodies (3). Although ≥65 years account for 61% of new cancers and 70% of deaths, they constituted only 25% of oncology trial participants, which is substantial underrepresentation (4).

For patients diagnosed with stage I to III NSCLC, surgical resection continues to be the primary treatment option, irrespective of age. Despite undergoing surgical resection, patients have a high risk of postoperative recurrence, ranging from 20% to 60% based on the disease stage (5).

Immune checkpoint inhibitors enhance antitumor immunity by blocking inhibitory signaling through checkpoint receptors on T lymphocytes and their ligands in tumor cells, has revolutionized the treatment of NSCLC. Perioperative immunotherapy has been approved as standard of care for resectable stage II-III NSCLC without driver mutations. However, T lymphocytes functions are subject to age-related alterations (6), which may contribute to differences in treatment outcomes between elderly and younger patients. The efficacy of perioperative immunotherapy in elderly patients remains unclear.

In this study, we undertook a systematic review and meta-analysis of clinical trials that focused on perioperative immunotherapy in aged ≥ 65 years resectable NSCLC patients. The efficacy and safety outcomes were evaluated across different groups.

## Methods

This systematic review and meta-analysis followed the Preferred Reporting Items for Systematic Reviews and Meta-analyses reporting guideline (7). The protocol was prospectively registered with PROSPERO (CRD420250654072).

### Data sources and search strategy

A comprehensive literature search was conducted across databases including PubMed, Embase, and proceedings of oncology conferences from January 1, 2020, to the present (search last updated June 30, 2025). Search parameters encompassed primary terms as PD - 1/PD-L1, neoadjuvant, adjuvant, perioperative, NSCLC.

### Inclusion criteria

Included studies (1) focused on patients with resectable NSCLC; (2) involved PD - 1 or PD-L1 inhibitors in neoadjuvant and/or adjuvant therapy; (3) compared groups receiving immunotherapy with placebo or chemo-immunotherapy with chemotherapy; (4) reported pCR, EFS and/or OS data in ≥65 years subgroup; and (5) were designed to be randomized clinical prospective studies.

### Data extraction and assessment of study quality

Two authors (Yue Cao and Yumeng Tian) independently extracted data and resolved discrepancies by consensus. Collected data pertained to outcomes including pCR, EFS, OS and its subgroup data. Ancillary details were recorded in the predefined information sheet. Methodological integrity was assessed using the Cochrane Risk of Bias Tool (8).

### Main outcomes

The co-primary outcomes encompass pCR, which is defined as the absence of residual invasive cancer on hematoxylin and eosin stained slides of the resected lung specimen and lymph nodes after the completion of neoadjuvant therapy, and EFS, which is defined as the duration from randomization to either local progression that impedes planned surgery, the presence of an unresectable tumor, disease progression or recurrence, or death in ≥65 years patients. The secondary outcome was OS in ≥65 years patients.

### Statistical analysis

Depending on the level of detail in the data disclosure for the elderly subgroup, the analysis encompasses three distinct strategies: (1) Neoadjuvant treatment regimen impacts the pathological response rate. Consequently, studies on neoadjuvant therapy were categorized and included in the analysis of pCR; (2) The studies that included neoadjuvant plus adjuvant therapy were considered for the EFS outcomes; (3) Due to the limitations of follow-up duration, the subgroup data regarding overall survival in the elderly population are likely constrained. Consequently, the data from the intention-to-treat (ITT) population were subjected to a meta-analysis, which was then compared with the disclosed data of the ≥65 years subgroup.

Initially, we performed direct meta-analysis comparing neoadjuvant chemoimmunotherapy with chemotherapy. The pooled relative risks (RRs) for pCR were derived via the Mantel-Haenszel method, whereas HRs for both EFS and OS were determined through the generic inverse-variance methods model. Intertrial heterogeneity was examined through the Cochran *Q* test, with *P* < .10 and *I* ^2^ > 50% demarcating significant heterogeneity—in such instances, a randomized effects model was used; otherwise, a fixed-effects model was applied (9).

For the subgroup analysis, we extracted relevant data from each study`s subgroups, pooled them through direct meta-analysis. Other statistical analyses were conducted with Review Manager software, version 5.4 (The Cochrane Collaboration). All tests were 2-sided, and a P value < .05 was considered significant unless otherwise indicated.

## Results

**Figure 1:**
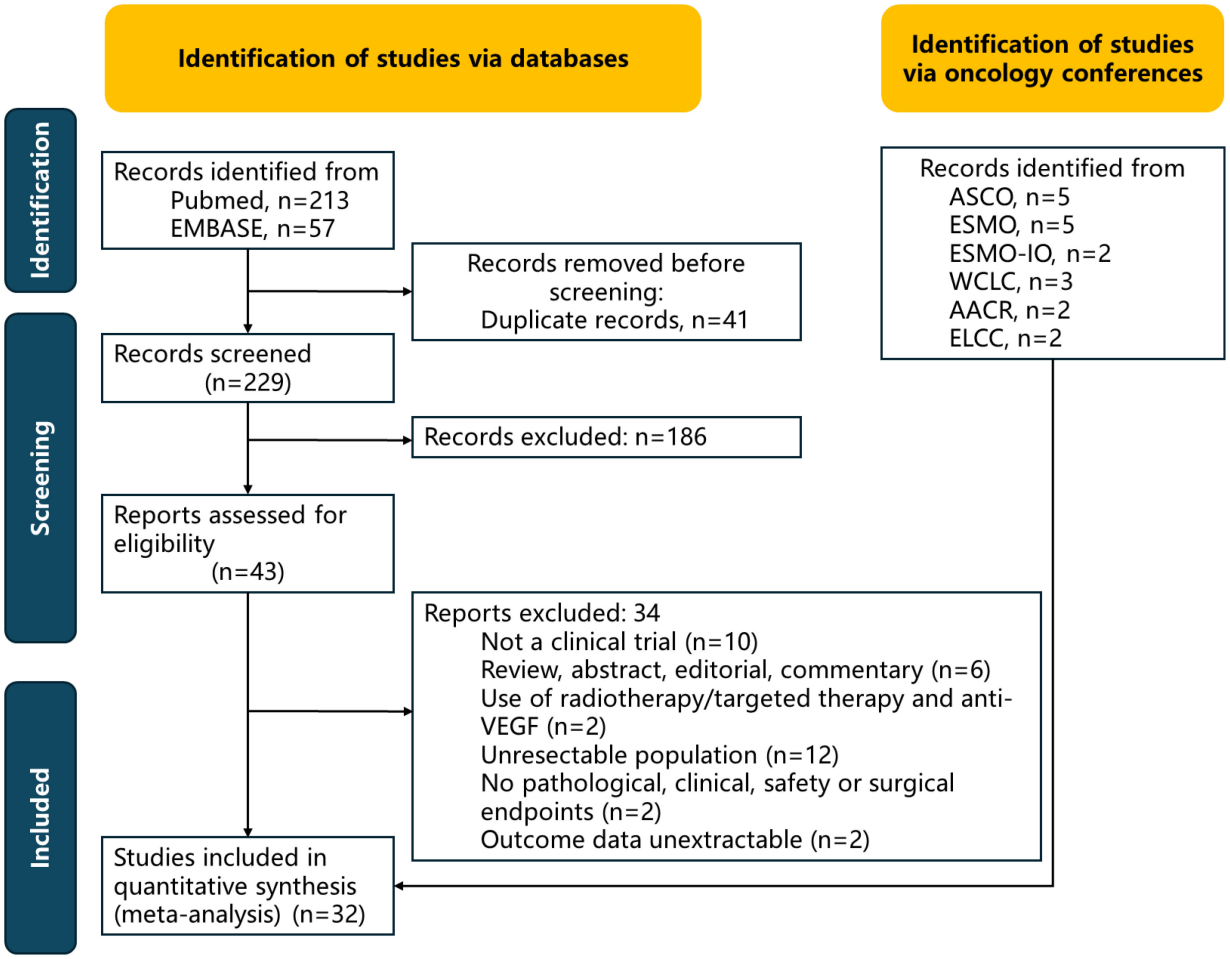
PRISMA diagram.

Eight randomized trials (3384 patients in total, including 1561 patients aged ≥65 years) were analyzed; the detailed selection flowchart is presented in **Figure 1**. Details of the bias assessment are provided in **Supplementary Table S1** and **Supplementary Figure S1**. A primary source of bias arose from the Neotorch study because short follow-up time.

### Trial characteristics

The characteristics and outcomes of the included trials are presented in **Table 1**. Six trials (AEGEAN, NADIM II, CheckMate 77T, KEYNOTE - 671, RATIONALE - 315 and Neotorch) explored perioperative immunotherapy vs placebo, while 2 trials (CheckMate 816 and TD-FOREKNOW) investigated neoadjuvant immunotherapy only.

**Table 1:** RCT study characteristics.

Treatment pattern	Identifier	Study name	Study phase	Study design	Neoadjuvant treatment regimen	Adjuvant treatment regimen	Main inclusion criteria	Primary endpoint	Patients, No.	Median age, yr	Maximum Age, yr	≥65 yr, %
Neoadjuvant	NCT02998528	CheckMate 816 (24–27)	III	Open label, randomized	Niovlumab + Platinum-doublet chemotherapy	/	Stage IB-IIIA resectable NSCLC per AJCC v7	pCR, EFS	179	64	82	48%
					Platinum-doublet chemotherapy	/			179	65	84	54%
	NCT04338620	TD-FOREKNOW (28)	II	Open label, randomized	Camrelizumab + Platinum-doublet chemotherapy	/	Stage IIIA-IIIB resectable NSCLC per AJCC v8	pCR	43	61	65	26%
					Platinum-doublet chemotherapy	/			45	61	65	31%
Neoadjuvant and adjuvant	NCT04025879	CheckMate 77T (29)	III	Double blind, randomized	Niovlumab + Platinum-doublet chemotherapy	Nivolumab	Stage IIA-IIIB N2 resectable NSCLC per AJCC v8	EFS	229	66	83	56%
					Platinum-doublet chemotherapy	Placebo			229	66	86	57%
	NCT03838159	NADIM II (30)	II	Open label, randomized	Nivolumab + Platinum-doublet chemotherapy	Nivolumab	Stage IIIA-IIIB resectable NSCLC per AJCC v8	pCR	57	65	70	49%
					Platinum-doublet chemotherapy	/			29	63	66	38%
	NCT03425643	KEYNOTE-671 (31–33)	III	Double blind, randomized	Pembrolizumab + Platinum-doublet chemotherapy	Pembrolizumab	Stage IIA-IIIB N2 resectable NSCLC per AJCC v8	EFS, OS	397	63	81	44%
					Platinum-doublet chemotherapy	Placebo			400	64	81	47%
	NCT04379635	RATIONALE-315 (34–36)	III	Double blind, randomized	Tislelizumab + Platinum-doublet chemotherapy	Tislelizumab	Stage II-IIIA N2 resectable NSCLC per AJCC v8	MPR, EFS	226	62	80	37%
					Platinum-doublet chemotherapy	Placebo			227	63	78	43%
	NCT04158440	Neotorch (stage III cohort) (37, 38)	III	Double blind, randomized	Toripalimab + Platinum-doublet chemotherapy	Toripalimab + Platinum-doublet chemotherapy for 1 cycle, then Toripalimab	Stage IIIA-IIIB N2 resectable NSCLC per AJCC v8	MPR, EFS	202	62	70	31%
					Platinum-doublet chemotherapy	Platinum-doublet chemotherapy for 1 cycle, then Placebo			202	61	70	32%
	NCT03800134	AEGEAN (39–42)	III	Double blind, randomized	Durvalumab + Platinum-doublet chemotherapy	Durvalumab	Stage IIA-IIIB N2 resectable NSCLC per AJCC v8	pCR, EFS	366	65	88	52%
					Platinum-doublet chemotherapy	Placebo			374	65	85	52%

Due to the different American Joint Committee on Cancer staging versions used during the study, Checkmate 816 enrolled patients with resectable NSCLC from stage IB to III according to the 7th edition, while other studies followed the 8th edition and enrolled patients with resectable NSCLC from stage II to III. Although the inclusion criteria of Neotorch included patients with stage II - III, only the data of the stage III cohort has been published so far.

In all experimental groups across these trials, 3 to 4 cycles of neoadjuvant PD - 1 or PD-L1 inhibitor plus platinum-based chemotherapy were administered. Predominantly, patients received a year of adjuvant immunotherapy, except in CheckMate 816 and TD-FOREKNOW. With a median follow-up time ranging from 18.3 to 57.6 months, the KEYNOTE - 671 and NADIM II study reported the OS data of the elderly patient subgroup.

### Comparison of pCR

**Figure 2:**
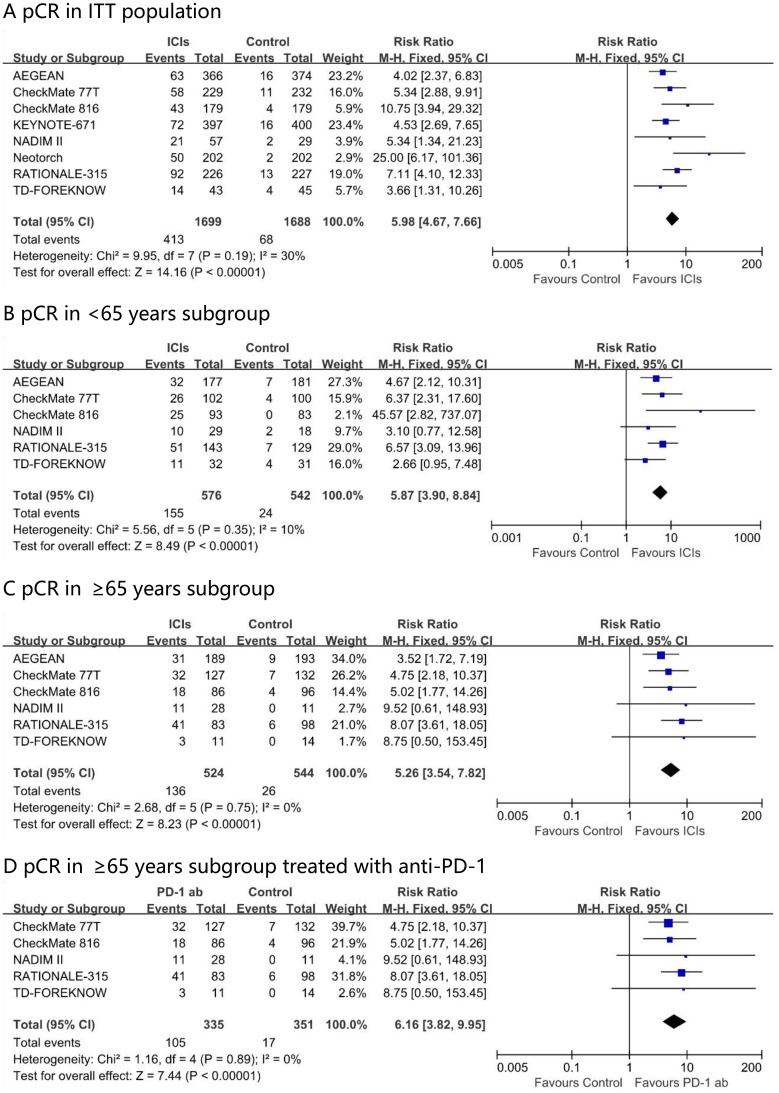
Pooled risk ratios of pCR in ITT population **(A)**, <65 years subgroup **(B)**, ≥65 years subgroup **(C)** and ≥65 years subgroup treated with anti-PD-1 **(D)** across randomized clinical trials.

In the neoadjuvant immunotherapy group, the pCR rate across the included trials ranged from 17.2% to 40.7%. Through direct meta-analysis, compared with chemotherapy alone, the addition of neoadjuvant immunotherapy was associated with a higher pCR rate (risk ratio, 5.98; 95% CI, 4.67 – 7.66; I² = 30%) in the intention-to-treat (ITT) population (**Figure 2**). This benefit was observed across all age subgroups, including those aged <65 years (risk ratio, 5.87; 95% CI, 3.60 – 8.84; I² = 10%) and those aged ≥65 years (risk ratio, 5.26; 95% CI, 3.54 – 7.82; I² = 0%). The advantage in terms of the pathological response rate was further enhanced in the PD - 1 antibody drug group for ≥65 years patients, with a risk ratio of 6.16 (95% CI, 3.82 – 9.95; I² = 0%).

### Comparison of EFS

**Figure 3:**
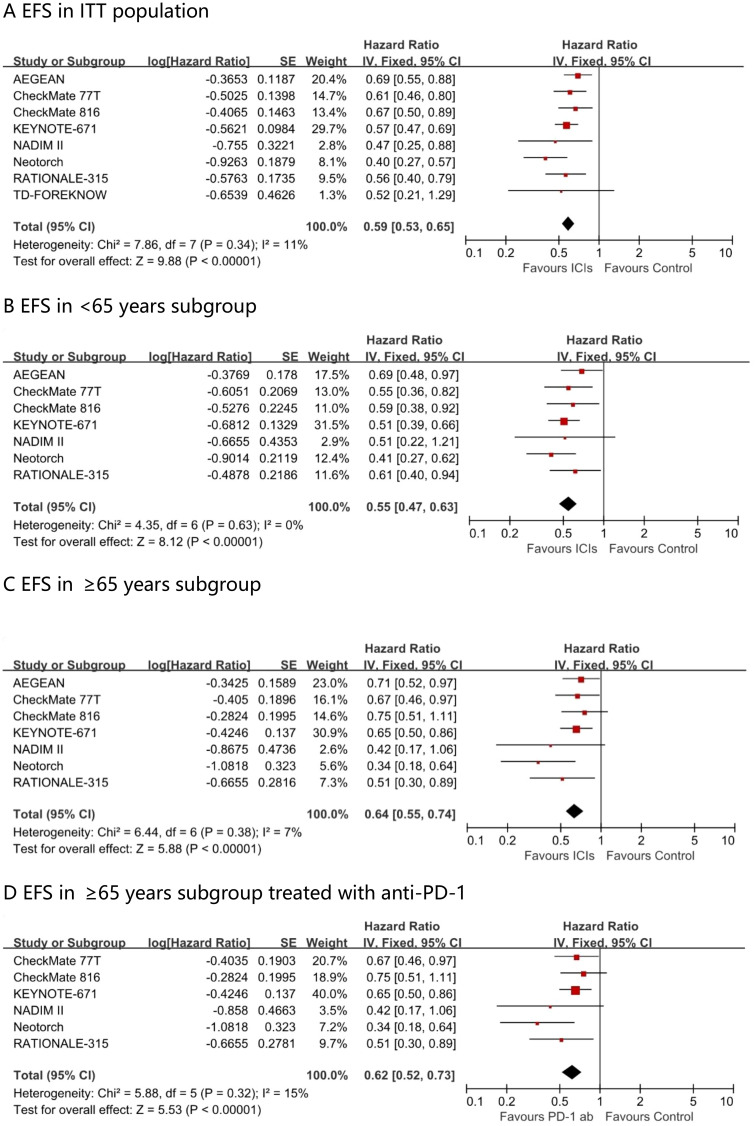
Pooled hazard ratios of event-free survival in ITT population **(A)**, <65 years subgroup **(B)**, ≥65 years subgroup **(C)** and ≥65 years subgroup treated with anti-PD-1 **(D)** across randomized clinical trials.

Fixed-effect meta-analysis estimated pooled EFS hazard ratios for perioperative immunotherapy versus placebo. A significant difference favoring perioperative immunotherapy over placebo was found in terms of EFS in the ITT population (HR, 0.59; 95% CI, 0.53 - 0.65; I² = 11%) (**Figure 3**). This benefit was observed across all age subgroups, including those aged <65 years (HR, 0.55; 95% CI, 0.47 – 0.63; I² = 0%) and those aged ≥65 years (HR, 0.64; 95% CI, 0.55 - 0.74; I² = 7%). The EFS advantage conferred by PD - 1 antibody in ≥65 years patients was comparable, with a hazard ratio of 0.62 (95% CI 0.52 – 0.73; I² = 15%).

### Comparison of OS

**Figure 4:**
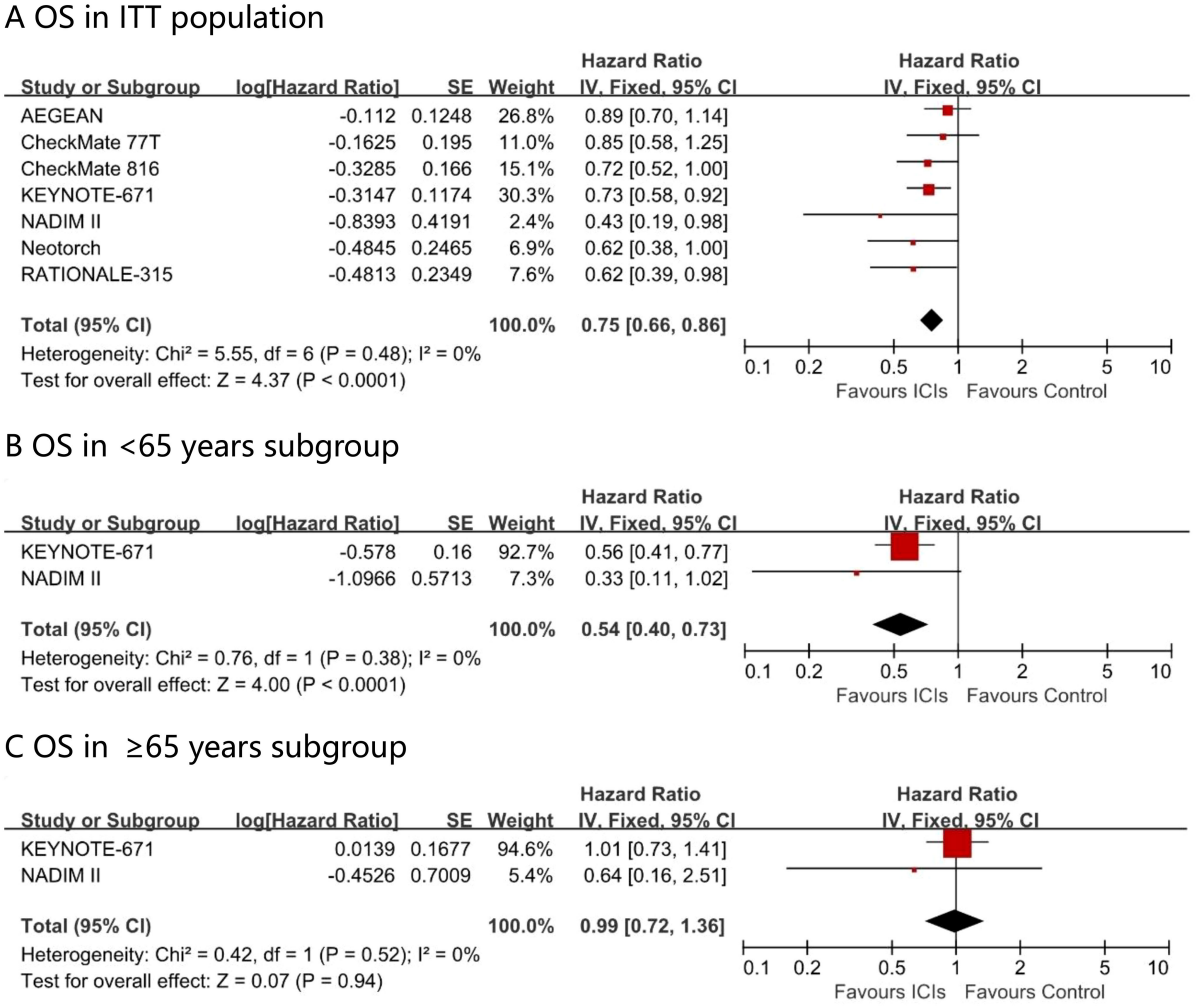
Pooled hazard ratios of overall survival in ITT population **(A)**, <65 years subgroup **(B)**, ≥65 years subgroup **(C)** and ≥65 years subgroup across randomized clinical trials.

Among the perioperative immunotherapy studies, seven trails (AEGEAN, CheckMate 77T, CheckMate 816, KEYNOTE - 671, NADIM II, Neotorch, and RATIONALE - 315) reported the overall survival (OS) in the intention-to-treat (ITT) population, with KEYNOTE - 671 and NADIM II providing OS data for the ≥ 65 years subgroup. A significant difference favoring perioperative immunotherapy over placebo was found in terms of OS in the ITT population (HR, 0.75; 95% CI, 0.66 - 0.86; I² = 0%) (**Figure 4**). Patients younger than 65 years gained a significant survival advantage (OS HR 0.54; 95% CI 0.40–0.73; I² = 0%), whereas no such benefit was observed in patients aged ≥65 years at the current follow-up time (OS HR 0.99; 95% CI 0.72–1.36; I² = 0%).

### Comparison of Surgical outcomes

**Figure 5:**
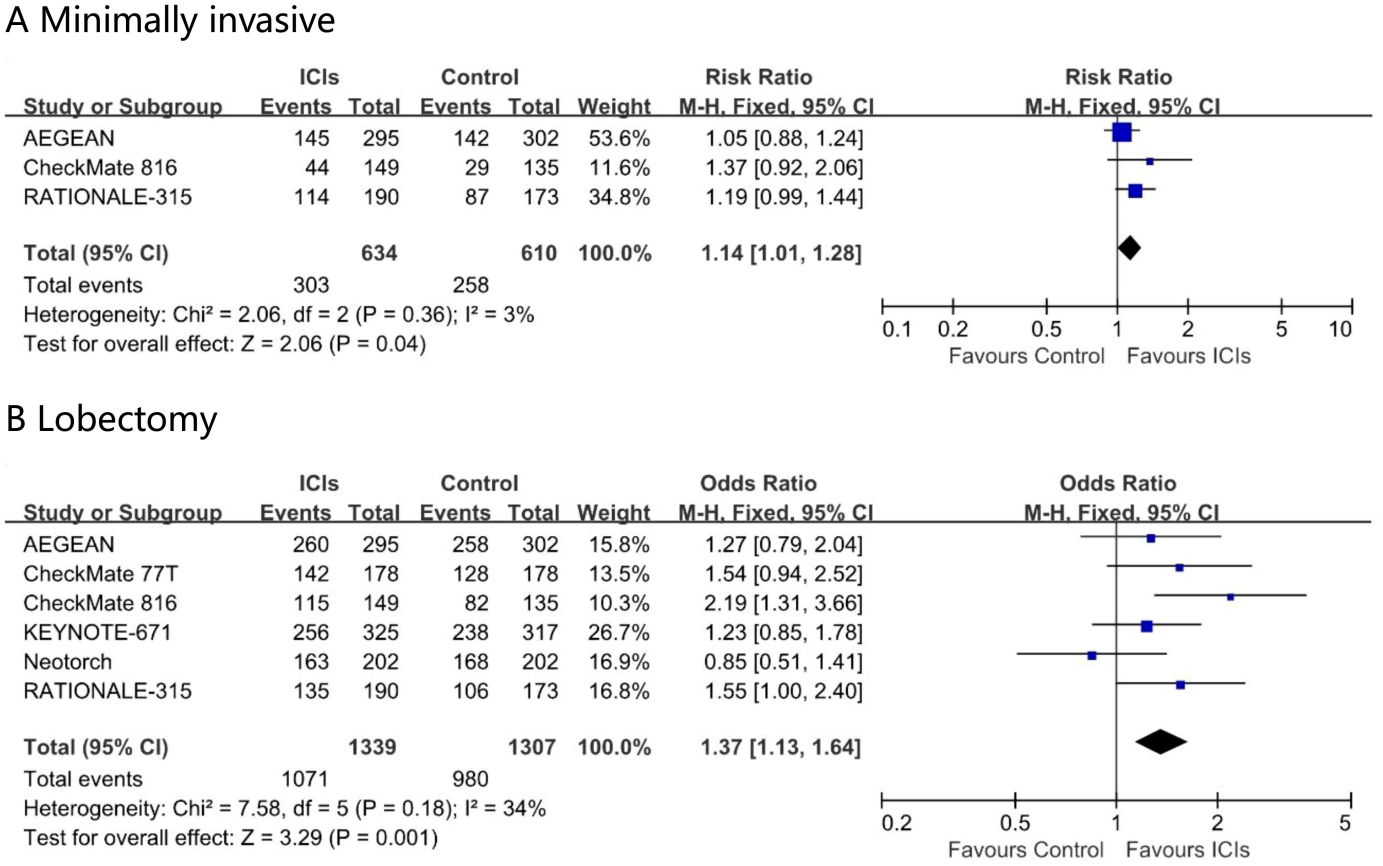
Pooled risk ratios of surgical outcomes, minimally invasive **(A)** and Lobectomy **(B)** in ITT population across randomized clinical trials.

Perioperative immunotherapy increased the rate of minimally invasive surgery (risk ratio, 1.13; 95% CI, 1.00 – 1.28; I² = 0%) and the proportion of lobectomy (risk ratio, 1.07; 95% CI, 1.01 – 1.14; I² = 58%) in the ITT population (**Figure 5**). None of the eight studies reported surgical outcome data specifically for the subgroup of patients aged 65 years or older.

### Comparison of treatment related adverse events

**Figure 6:**
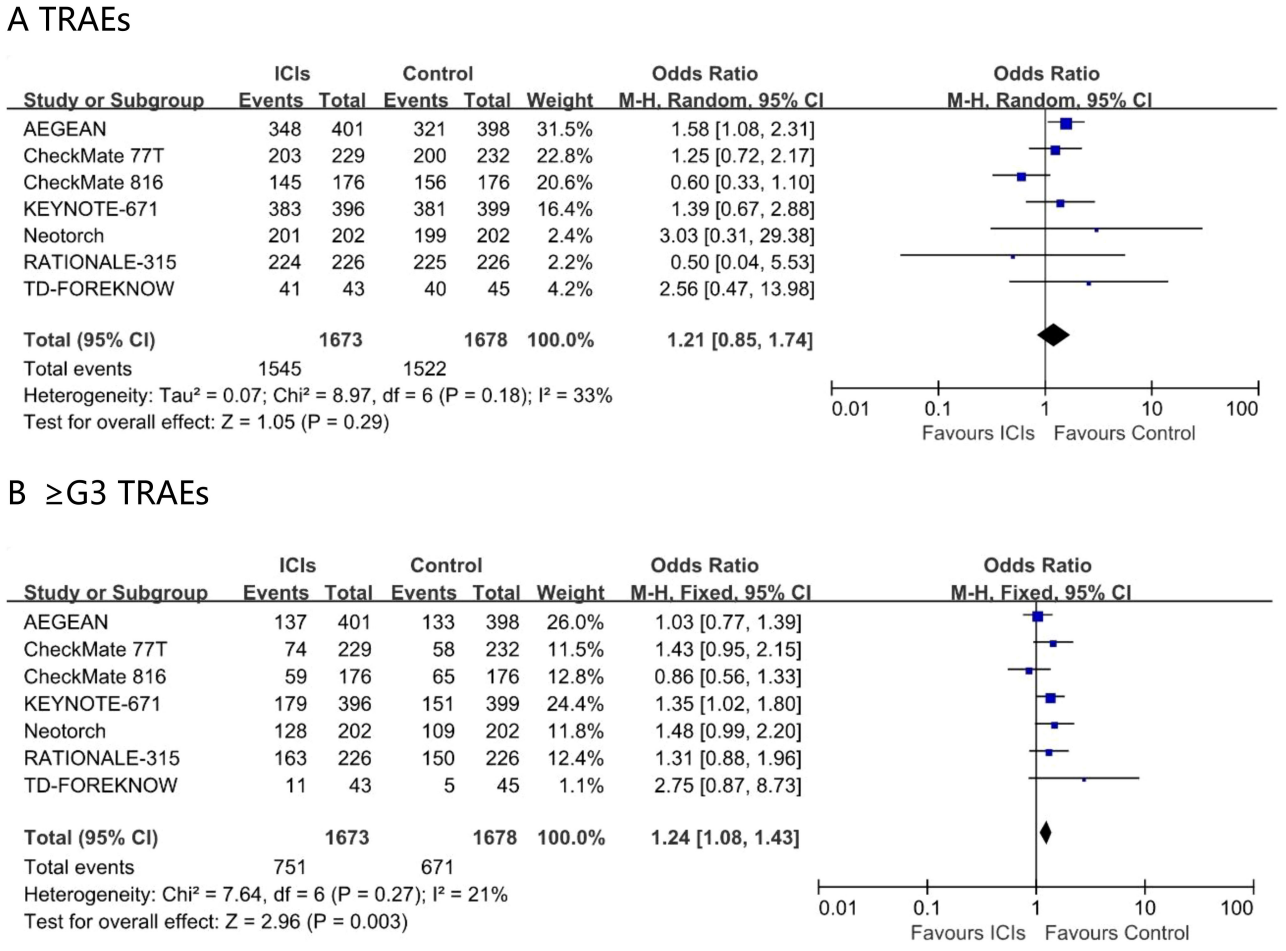
Pooled risk ratios of TRAEs **(A)**, ≥G3 TRAEs **(B)** in ITT population across randomized clinical trials.

In the ITT population, the incidence of TRAEs did not differ significantly between perioperative immunochemotherapy and chemotherapy alone (odds ratio 1.21; 95% CI 0.85 – 1.74; I² = 23%). Nevertheless, the addition of immunotherapy conferred a modest yet statistically significant increase in grade ≥3 TRAEs (odds ratio 1.24; 95% CI 1.08 – 1.43; I² = 21%) (**Figure 6**). Safety outcomes stratified by age were not reported.

## Discussion

To our knowledge, our meta-analysis is the first to show that adding perioperative PD - 1 or PD-L1 antibodies improves pCR and EFS in elderly patients with stage II-III resectable NSCLC. Moreover, the trend of benefit for elderly patients aligns with that of the ITT population and the <65 years subgroup, and increasing age does not diminish this advantage. Nevertheless, although an overall survival advantage was evident in the ITT population, this benefit was not replicated in the elderly subgroup.

Research by the British Thoracic Association indicates that advanced age is not a contraindication for surgery (10). In elderly early-stage NSCLC patients, the overall survival rate and lung cancer specific survival rate after surgery are higher than with radiotherapy or no treatment, and surgery may offer greater potential survival benefits for elderly NSCLC patients (11). Among the factors influencing surgery, age ranks relatively low. Age alone does not preclude patients from undergoing surgery. Comorbidities, surgical approach, and the extent of resection have a more significant impact on surgical decision-making in elderly patients. Minimally invasive surgery (12), and lobectomy (13) are more suitable for elderly patients. Perioperative immunotherapy increased the rate of minimally invasive surgery and the proportion of lobectomy, which is a positive sign for curative-intent therapy.

As elderly patients often exhibit diminished tolerance to conventional anticancer therapies, immune checkpoint inhibitors—characterized by a more favorable toxicity profile—offer renewed therapeutic option for this vulnerable population. In resectable NSCLC, CheckMate 159 (14) and CheckMate 816 (15) offer two neoadjuvant paradigm, mono-immunotherapy and chemo-immunotherapy, demonstrate that neoadjuvant single-agent immunotherapy still confers meaningful improvements in pCR EFS and OS. Nevertheless, when platinum-based chemotherapy is contraindicated by age-related frailty or comorbidity, peri-operative single-agent immunotherapy remains a clinically feasible and well-tolerated option.

Regarding treatment modalities, both neoadjuvant-only and perioperative immunotherapy have yielded positive results, but it remains to be determined which approach is superior. The individual patient level data analysis of CheckMate 77T and CheckMate 816 shows that perioperative nivolumab demonstrated an improvement in EFS compared to neoadjuvant nivolumab alone in patients with resectable NSCLC (16). In our study, the perioperative EFS (HR 0.62, **Supplementary Figure S2**) from the meta-analysis of the elderly subgroup was superior to that from Checkmate 816 (HR 0.75) (15), which is consistent with previous findings.

We also observe that anti-PD-1 demonstrates improved pCR rate compared with anti-PD-L1. In terms of molecular mechanisms, PD - 1 antibodies bind to PD - 1 and simultaneously block the binding of PD - 1 to its ligands (PD-L1 and PD-L2). However, while PD-L1 antibodies inhibit the binding of PD - 1 to PD-L1, the interaction between PD - 1 and PD-L2 remains intact, potentially inhibiting T cell activation. Thus, tumors may escape antitumor immune responses through the PD - 1/PD-L2 axis when treated with anti-PD-L1.

In the ITT population, the incidence of TRAEs was comparable between perioperative chemo-immunotherapy and chemotherapy alone; however, grade ≥3 TRAEs were more frequent with chemo-immunotherapy. Since the randomized controlled trial did not conduct an age-based subgroup analysis for adverse events, it was not possible to perform a meta-analysis. However, we have identified some data from real-world studies. Liu et al. retrospective compared the efficacy and safety of neoadjuvant immunochemotherapy in young and elderly patients with IIA-IIIB NSCLC in real world practice and found that the incidence of AEs was similar in young and elderly patients (17). Muhammad et al. retrospectively analyzed 19177 cancer patients and found that elderly recipients of immune checkpoint inhibitors are not at increased risk of irAE compared to younger patients (18). In parallel, three retrospective analyses reported that elderly patients experience higher incidences of dermatologic, pulmonary, and gastrointestinal immune-related adverse events during checkpoint inhibitor therapy (19–21).

Notably, immunotherapy yielded a favorable EFS yet unfavorable OS in elderly patients; several factors may explain this paradox. Firstly, elderly patients were not a predefined subgroup, which may lead to baseline imbalance. Secondly, death events in elderly patients are caused by multiple factors, with a higher incidence of non-tumor-related mortality. Using nationwide Japanese registry data, Yasufumi et al. found that non-cancer mortality rose to 31.6% by 4 years since diagnosis, driven mainly by heart (21.8%) and cerebrovascular disease (9.8%); both are markedly more common in the elderly cancer patients (22). Additionally, immune senescence impairs both innate and adaptive immunity: naive T/B cells drop while memory subsets rise, dendritic cell and natural killer cell functions decline, yet myeloid-derived suppressor cells and macrophages expand and hyper-function (23). Together, these changes may blunt the efficacy of immune-checkpoint inhibitors in elderly cancer patients. Therefore, lung cancer-specific survival may serve as a more precise endpoint for anti-tumor therapy research in elderly patients.

### Limitations

This study had several limitations that make any recommendations preliminary. First, given the inclusion and exclusion criteria of the randomized controlled trial, the study involved elderly patients who were fit for surgical resection and chemoimmunotherapy, typically those with better physical fitness. As a result, the study’s findings are mainly applicable to this specific group of patients. Second, the follow-up duration in our study was relatively short, and only two studies reported the overall survival data for elderly patients aged 65 and above, so we could not clearly determine the long-term efficacy in elderly NSCLC patients. Third, the safety meta-analysis investigating the incidence of adverse events in elderly patients aged 65 and above was not conducted, due to insufficient original literature data.

## Conclusions

In summary, this meta-analysis concluded that perioperative immunotherapy was superior to placebo in terms of pathological and efficacy outcomes for patients aged ≥65 years. Elderly patients derived benefits in terms of pathological complete response and event-free survival with perioperative immunotherapy. The study has spotted positive signs of perioperative immunotherapy for elderly patients with resectable stage II-III NSCLC. However, further prospective studies are needed to address this clinical question.
