# Supplementary material for: Perioperative immune checkpoint inhibitors in elderly patients with resectable NSCLC: a systematic review and meta-analysis
Source: Front Oncol. 2025 Sep 17;15:1589846. doi: 10.3389/fonc.2025.1589846 (PMC12483896; doi:10.3389/fonc.2025.1589846)

Table S1. Quality assessment by cochrane collaboration`s Tool

Trial	Sequence generation	Allocation concealment	Blinding	Incomplete outcome data	Selectvie reporting	Other source of bias
CheckMate 816	Adequate	Adequate (Central allocation)	Open label	Adequate	Adequate	
NADIM II	Adequate	Adequate	Open label	Adequate*	Adequate	
TD-FOREKNOW	Adequate	Adequate	Open label	Adequate*	Adequate	
CheckMate 77T	Adequate	Adequate (Central allocation)	Double blinded	Adequate*	Adequate	
KEYNOTE-671	Adequate	Adequate (Central allocation)	Double blinded	Adequate*	Adequate	
RATIONALE-315	Adequate	Adequate (Central allocation)	Double blinded	Adequate*	Adequate	
Neotorch (stage III cohort)	Adequate	Adequate (Central allocation)	Double blinded	Adequate*	Adequate	Short follow-up duration
AEGEAN	Adequate	Adequate (Central allocation)	Double blinded	Adequate*	Adequate	

*data concerning event-free survival were adequate, while date of overall survival has yet to mature in the trial.

Figure S1 Funnel plots of publication bias

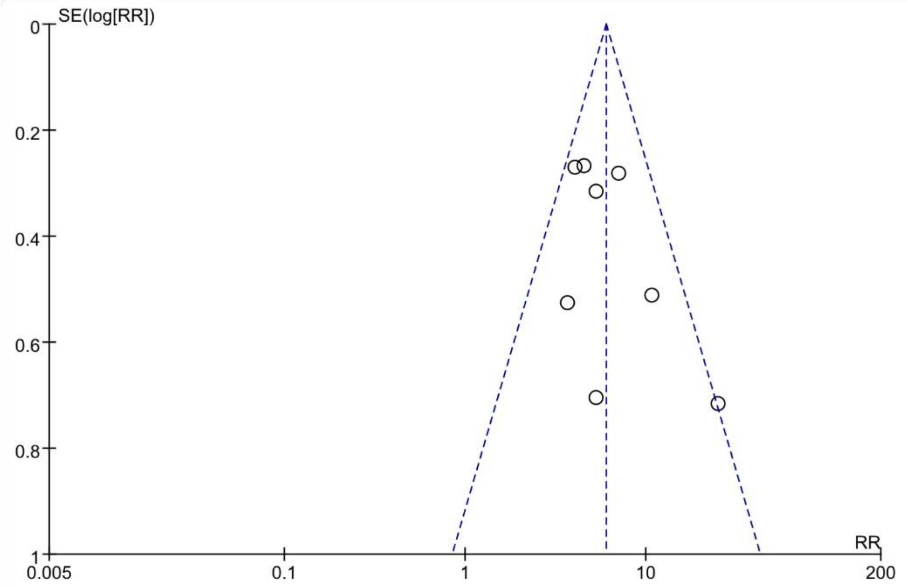

Figure S2 Pooled hazard ratios of event-free survival in ≥65 years patients of perioperative immunotherapy across randomized clinical trial

Study or Subgroup	log[Hazard Ratio]	SE	Weight	Hazard Ratio IV, Fixed, 95% CI
AEGEAN	-0.3425	0.1589	26.9%	0.71 [0.52, 0.97]
CheckMate 77T	-0.405	0.1896	18.9%	0.67 [0.46, 0.97]
KEYNOTE-671	-0.4246	0.137	36.2%	0.65 [0.50, 0.86]
NADIM II	-0.8675	0.4736	3.0%	0.42 [0.17, 1.06]
Neotorch	-1.0818	0.323	6.5%	0.34 [0.18, 0.64]
RATIONALE-315	-0.6655	0.2816	8.6%	0.51 [0.30, 0.89]
**Total (95% CI)**			**100.0%**	**0.62 [0.53, 0.73]**

Heterogeneity: Chi² = 5.64, df = 5 (P = 0.34); I² = 11%
Test for overall effect: Z = 5.77 (P < 0.00001)

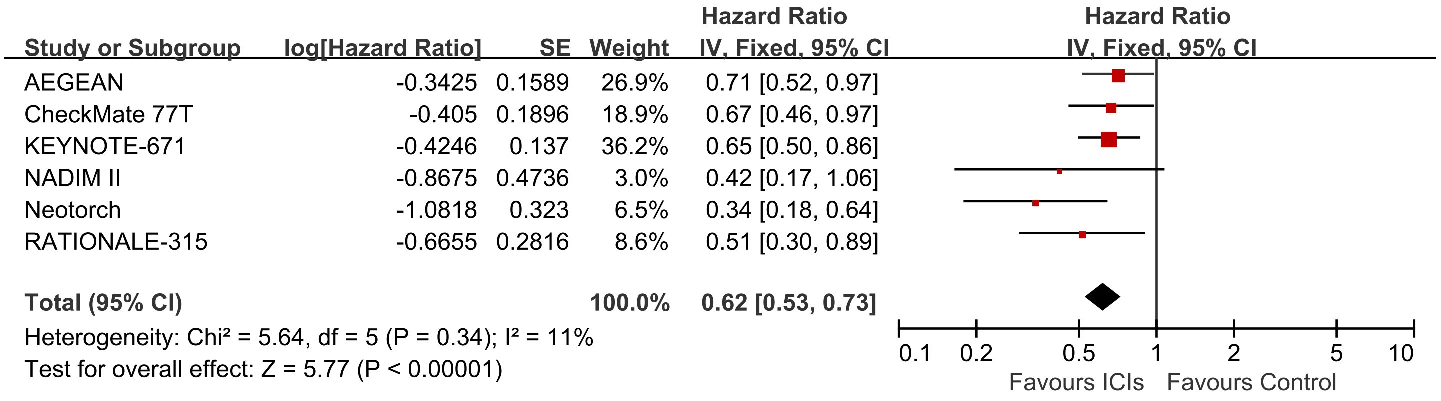

Supplement: Supplementary file 1 [file DataSheet1.pdf]
